# The complete genome sequence of the rumen bacterium *Butyrivibrio hungatei* MB2003

**DOI:** 10.1186/s40793-017-0285-8

**Published:** 2017-12-04

**Authors:** Nikola Palevich, William J. Kelly, Sinead C. Leahy, Eric Altermann, Jasna Rakonjac, Graeme T. Attwood

**Affiliations:** 1 Rumen Microbiology, Animal Science, AgResearch Limited, Grasslands Research Centre, Tennent Drive, Private Bag 11008, Palmerston North, 4442 New Zealand; 2 Institute of Fundamental Sciences, Massey University, Palmerston North, New Zealand

**Keywords:** Rumen, Bacteria, Hemicellulose, Pectin, Degradation, *Butyrivibrio*, Genome

## Abstract

*Butyrivibrio hungatei* MB2003 was isolated from the plant-adherent fraction of rumen contents from a pasture-grazed New Zealand dairy cow, and was selected for genome sequencing in order to examine its ability to degrade plant polysaccharides. The genome of MB2003 is 3.39 Mb and consists of four replicons; a chromosome, a secondary chromosome or chromid, a megaplasmid and a small plasmid. The genome has an average G + C content of 39.7%, and encodes 2983 putative protein-coding genes. MB2003 is able to use a variety of monosaccharide substrates for growth, with acetate, butyrate and formate as the principal fermentation end-products, and the genes encoding these metabolic pathways have been identified. MB2003 is predicted to encode an extensive repertoire of CAZymes with 78 GHs, 7 CEs, 1 PL and 78 GTs. MB2003 is unable to grow on xylan or pectin, and its role in the rumen appears to be as a utilizer of monosaccharides, disaccharides and oligosaccharides made available by the degradative activities of other bacterial species.

**Electronic supplementary material:**

The online version of this article (10.1186/s40793-017-0285-8) contains supplementary material, which is available to authorized users.

## Introduction

10.1601/nm.4129 are important rumen bacteria [1], and are among the small number of rumen genera capable of utilizing the complex plant structural polysaccharides xylan and pectin [2, 3]. They are classified as anaerobic, monotrichous, butyrate-producing, curved rods and have been isolated from the gastrointestinal tracts and feces of various ruminants, monogastric animals and humans [4, 5]. 10.1601/nm.4129 are metabolically versatile and are capable of growing on a range of carbohydrates, from simple mono- or oligosaccharides to complex plant polysaccharides such as pectins, mannans, starch and hemicelluloses [6]. Furthermore, xylans of diverse chemical and physical properties, from a range of forages are degraded by 10.1601/nm.4129 species [7]. Some 10.1601/nm.4129 species show strong proteolytic activity [8], and 10.1601/nm.4129 are thought to be the main butyrate producers in the rumen [9, 10]. The genus 10.1601/nm.4129 is classified within the family 10.1601/nm.4118, order 10.1601/nm.17931, and is phylogenetically diverse. The 10.1601/nm.4129 genus originally consisted of only one species, 10.1601/nm.4130 [2]. In addition to phenotypic characterisations [11, 12], studies have utilized DNA-DNA hybridization [13, 14], 16*S* rRNA gene sequencing [15, 16] and 16*S* rRNA-based hybridization probes [17], to differentiate these organisms. To accommodate the observed diversity amongst the newly discovered bacterial strains, a new genus, 10.1601/nm.4145, was described [18]. Four species are currently recognized: 10.1601/nm.4130, 10.1601/nm.4132, 10.1601/nm.13010 and 10.1601/nm.4131 [6], although 10.1601/nm.4131 is more distantly related to the other three. 10.1601/nm.4132 are common anaerobic rumen bacteria found in domestic and wild ruminants and the type strain is JK615^T^ [19]. 10.1601/nm.4132 JK615^T^ is non-proteolytic and non-fibrolytic, but is able to utilize oligo- and monosaccharides as substrates for growth. Gaining an insight into the role of these secondary degrader species in microbial plant polysaccharide breakdown is important for understanding rumen function. Here we present the complete genome sequence of 10.1601/nm.4132 MB2003, a strain isolated from a pasture-grazed dairy cow in New Zealand [20], and describe its comparison with genomes of closely related 10.1601/nm.4132 strains.

## Organism information

### Classification and features

**Fig. 1:**
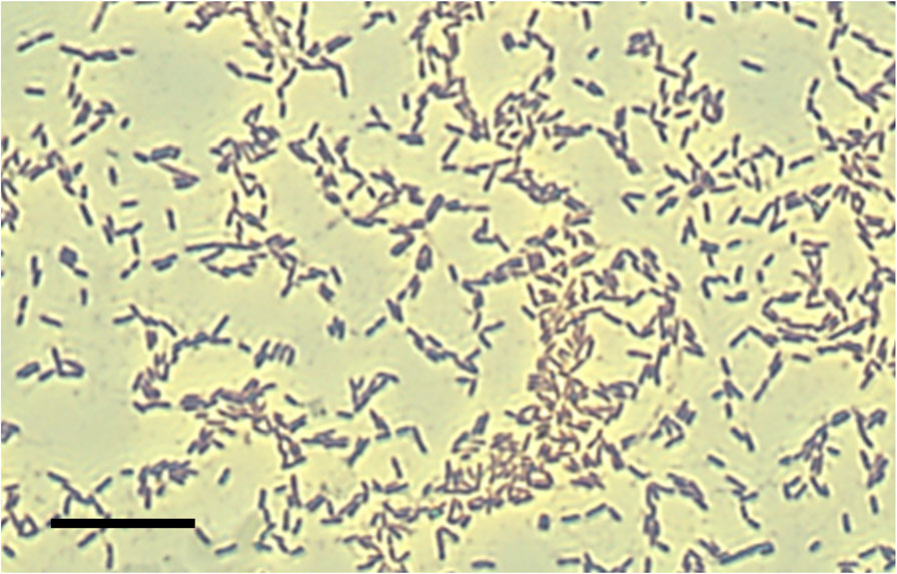
Morphology of *B. hungatei* MB2003. Micrograph of Gram stained *B. hungatei* MB2003 cells at 100 × magnification. Bar represents 10 μm

**Fig. 2:**
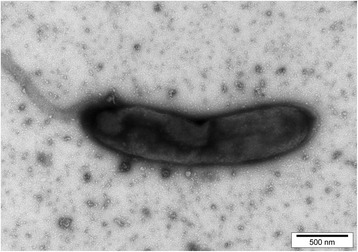
Transmission electron micrograph of *B. hungatei* MB2003. Micrograph of negatively stained *B. hungatei* MB2003 cells at 10,000 × magnification

**Fig. 3:**
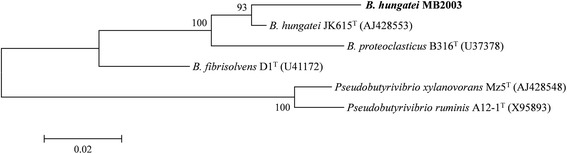
Phylogenetic tree highlighting the relationship of *B. hungatei* MB2003 relative to the type strains of the other species within the genus *Butyrivibrio*. The evolutionary history was inferred using the Maximum Likelihood method based on the General Time Reversible model [55]. The tree with the highest log likelihood (−3712.3329) is shown. The percentage of replicate trees in which the associated taxa clustered together in the bootstrap test (10,000 replicates) is shown next to the branches [56]. Initial tree(s) for the heuristic search were obtained automatically by applying Neighbor-Join and BioNJ algorithms to a matrix of pairwise distances estimated using the Maximum Composite Likelihood (MCL) approach, and then selecting the topology with superior log likelihood value. A discrete Gamma distribution was used to model evolutionary rate differences among sites (5 categories (+G, parameter = 0.3950)). The tree is drawn to scale, with branch lengths measured in the number of substitutions per site. The analysis involved six nucleotide sequences. All positions with less than 95% site coverage were eliminated. There were a total of 1509 positions in the final dataset. Evolutionary analyses were conducted in MEGA6 [55]. GenBank accession numbers of the 16*S* rRNA gene sequences are shown in parentheses. Bar, 0.02 nucleotide substitutions per site. ^T^, indicates type strain. All the type strains have their genome sequencing projects registered in the Genomes Online Database (GOLD) [57]

MB2003 was isolated from the plant-adherent fraction of rumen contents from a New Zealand dairy cow grazing fresh forage [20, 21]. MB2003 cells are Gram positive, short rods, occurring singly or in pairs (Fig. 1). The morphological features of MB2003 cells were determined by electron microscopy of cells grown on RM02 medium [22], negatively stained with 1% phosphotungstic acid, mounted on Formvar-coated copper grids, and examined using a Philips model 201C electron microscope (Eindhoven, The Netherlands). MB2003 cells were observed to have a single polar flagellum (Fig. 2), although cells in growing cultures were non-motile. A phylogenetic analysis of the full-length 16*S* rRNA gene sequence placed MB2003 within the 10.1601/nm.4132 species, being 98% similar to the 10.1601/nm.4132 type strain JK615^T^ [19] (Fig. 3). Additional characteristics of 10.1601/nm.4132 MB2003 are shown in Table 1.

**Table 1:** Classification and general features of the rumen bacterium *B. hungatei* MB2003 in accordance with the MIGS recommendations [58]

MIGS ID	Property	Term	Evidence code^a^
	Current classification	Domain: *Bacteria*	TAS [59]
		Phylum: *Firmicutes*	TAS [60, 61]
		Class: *Clostridia*	TAS [62]
		Order: *Eubacteriales*	TAS [63]
		Family: *Lachnospiraceae*	TAS [64]
		Genus: *Butyrivibrio*	TAS [4]
		Species: *hungatei*	TAS [19]
		Type strain: No	
		Strain: MB2003	TAS [20, 21]
	Gram stain	Positive	TAS [21, 31]
	Cell shape	Rod	TAS [21, 31]
	Motility	Non-motile	IDA
	Sporulation	Not reported	NAS
	Temperature range	37–39 °C	IDA
	Optimum temperature	39 °C	IDA
	pH range; Optimum	6.0–7.0; 6.4	IDA
	Carbon source	Variety of carbohydrates	IDA
	Energy metabolism	Fermentative metabolism	IDA
MIGS-6	Habitat	Bovine rumen	TAS [20]
MIGS-6.3	Salinity	Not reported	
MIGS-22	Oxygen requirement	Anaerobic	IDA
MIGS-15	Biotic relationship	Symbiont of ruminants	TAS [20]
MIGS-14	Pathogenicity	Non-pathogen	NAS
MIGS-4	Geographic location	Ruakura, Hamilton, New Zealand	TAS [20]
MIGS-5	Sample collection time	May 2009	TAS [20]
MIGS-4.1	Latitude	−37.77 (37°46′28″S)	IDA
MIGS-4.2	Longitude	+175.31 (175°18′31″E)	IDA
MIGS-4.4	Altitude	40 m	IDA

^a^Evidence codes - IDA, Inferred from Direct Assay, NAS, Non-traceable Author Statement (i.e., not directly observed for the living, isolated sample, but based on a generally accepted property for the species, or anecdotal evidence). Evidence codes are from the Gene Ontology project [65]

**Fig. 4:**
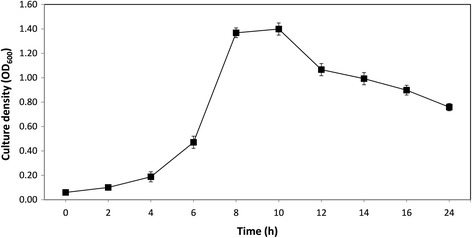
Culture density achieved in 24 h by MB2003 growing in media with cellobiose as the sole substrate. Points indicate means of three replicates, and the error bars represent +/−one standard error

Strain MB2003 grew to highest optical density (OD) at pH values of 6.1 to 6.5 and at a temperature of 39 °C, conditions which are typical of its rumen environment. VFA production was determined from triplicate broth cultures grown overnight in RM02 medium with cellobiose as substrate and analysed for formate, acetate, propionate, *n*-butyrate, *iso*-valerate and lactate on a HP 6890 series GC (Hewlett Packard) with 2-ethylbutyric acid (Sigma-Aldrich, St. Louis, MO, USA) as the internal standard. To derivatize formic, lactic and succinic acids, samples were mixed with HCl ACS reagent (Sigma-Aldrich, St. Louis, MO, USA) and diethyl ether, with the addition of *N*-methyl-*N*-*t*-butyldimethylsilyltri-fluoroacetamide (MTBSTFA) (Sigma-Aldrich, St. Louis, MO, USA) [23]. Under these conditions MB2003 produced 16.4 mM formate, 3.6 mM acetate and 4.7 mM butyrate. MB2003 was able to grow in CO_2_-containing media with various soluble carbon sources and the semi-soluble inulin (all tested at 0.5% *w*/*v* final concentration). Growth on soluble substrates was assessed as an increase in culture density OD_600nm_ compared to cultures without carbon source added, whereas total VFA production was used as an indicator of substrate utilization and growth for insoluble polymers (Table 2). All strains tested were net producers of formate, acetate and *n*-butyrate, which is characteristic of 10.1601/nm.4129 *.* Cellobiose and glucose supported the growth of MB2003, JK615^T^ and B316^T^ to high cell densities. Therefore, cellobiose was used to examine the growth of MB2003 over a 24 h period. The exponential phase of growth was between 4 and 8 h, with the maximum cell density reached at 8 to 10 h, and stationary phase between 10 to 24 h (Fig. 4).

**Table 2:** Carbon source utilization of the *Butyrivibrio* strains

Substrate		MB2003	JK615^T^	B316^T^
Monosaccharides	Arabinose	++	++	++
	Fructose	–	–	++
	Galactose	++	–	++
	Glucose	++	++	++
	Mannose	–	++	++
	Rhamnose	–	–	++
	Ribose	–	–	–
	Xylose	++	++	++
Disaccharides	Cellobiose	++	++	++
	Lactose	++	++	++
	Maltose	++	++	++
	Melibiose	–	–	+
	Sucrose	++	++	++
Trisaccharides	Melezitose	–	–	++
	Raffinose	–	++	++
	Trehalose	–	–	++
Sugar Alcohols	myo-Inositol	–	–	–
	Mannitol	–	–	+
	Sorbitol	–	–	–
Glycosides	Amygdalin	+	–	++
	Esculin	–	++	++
	Rutin	–	++	++
	Salicin	++	++	++
Insoluble substrates	Cellulose	–	–	–
	Dextrin	–	–	++
	Inulin	+	–	++
	Starch	–	–	++
	Pectin	–	–	++
	Xylan	–	–	++

ΔOD_600nm_ readings of 0.5–1.0 were scored as ++, 0.2–0.5 scored as +, and 0–0.2 scored as -. Results for *B. hungatei* JK615^T^ and *B. proteoclasticus* B316^T^ are adapted from Kopečný et al. [19] and Moon et al. [6], respectively

## Genome sequencing information

### Genome project history

10.1601/nm.4132 MB2003 was selected for genome sequencing as a NZ strain of 10.1601/nm.4132. A summary of the genome project information is shown in Table 3 and in Additional file 1: Table S1.

**Table 3:** MB2003 genome project information

MIGS ID	Property	Term
MIGS-31	Finishing quality	High-quality, closed genome
MIGS-28	Libraries used	454 3 kb mate paired-end library
MIGS-29	Sequencing platforms	454 GS FLX Titanium chemistry
MIGS-31.2	Fold coverage	234×
MIGS-30	Assemblers	Newbler version 2.3
MIGS-32	Gene calling method	Glimmer and BLASTX
	Locus Tag	bhn and bhn_RS
	Genbank ID	CP017830, CP017831, CP017832, CP017833
	Genbank Date of Release	31 October 2016
	GOLD ID	Ga0074201
	BIOPROJECT ID	PRJNA349214 and PRJNA224116
	BIOSAMPLE ID	SAMN05928573
MIGS-13	Source Material Identifier	*Butyrivibrio hungatei* MB2003
	Project relevance	Ruminant plant-fibre degradation

### Growth conditions and genomic DNA preparation

MB2003 was grown in RM02 medium [22] with 10 mM glucose and 0.1% yeast extract but without rumen fluid. Culture purity was confirmed by Gram stain and sequencing of the 16*S* rRNA gene. Genomic DNA was extracted from freshly grown cells by a modification of the standard cell lysis method of Saito and Miura [24], using lysozyme, proteinase K and sodium dodecyl sulphate, followed by phenol-chloroform extraction, and purification using the Qiagen Genomic-Tip 500 Maxi kit (Qiagen, Hilden, Germany). Genomic DNA was precipitated by the addition of a 0.7 volume of isopropanol, and collected by centrifugation at 12,000×*g* for 10 min at room temperature. The supernatant was removed, and the DNA pellet was washed in 70% ethanol, re-dissolved in TE buffer (10 mM Tris-HCl, 1 mM EDTA, pH 7.5) and stored at −20 °C until required.

### Genome sequencing and assembly

The complete genome sequence of MB2003 was determined by pyrosequencing 3 kb mate paired-end sequence libraries using the 454 GS FLX platform with Titanium chemistry (Macrogen, Korea). Pyrosequencing reads provided 234× coverage of the genome and were assembled using the Newbler assembler (version 2.7, Roche 454 Life Sciences, USA) which resulted in 31 contigs across 7 scaffolds. Gap closure was managed using the Staden package [25] and gaps were closed using additional Sanger sequencing by standard and inverse PCR techniques. In addition, MB2003 genomic DNA was sequenced using shotgun sequencing of 2 kb paired-end sequence libraries using the Illumina MiSeq platform (Macrogen, Korea) which provided 800-fold sequencing coverage. Illumina reads were analysed using the Galaxy web-based platform [26] and de novo assembly was performed using the Velvet assembler, version 3.0 [27]. The Velvet assembled MB2003 genome MiSeq sequences were combined with the Newbler assembly using the Staden package and Geneious, version 8.1 [28]. Genome assembly was confirmed by pulsed-field gel electrophoresis.

### Genome annotation

Annotation of the MB2003 genome was performed as described previously [29]. The MB2003 genome sequence was prepared for NCBI submission using Sequin [30], and the adenine residue of the start codon of the chromosomal replication initiator protein DnaA1 (bhn_I0001, bhn_RS00450) gene was chosen as the first base for the MB2003 genome.

### Genome properties

**Fig. 5:**
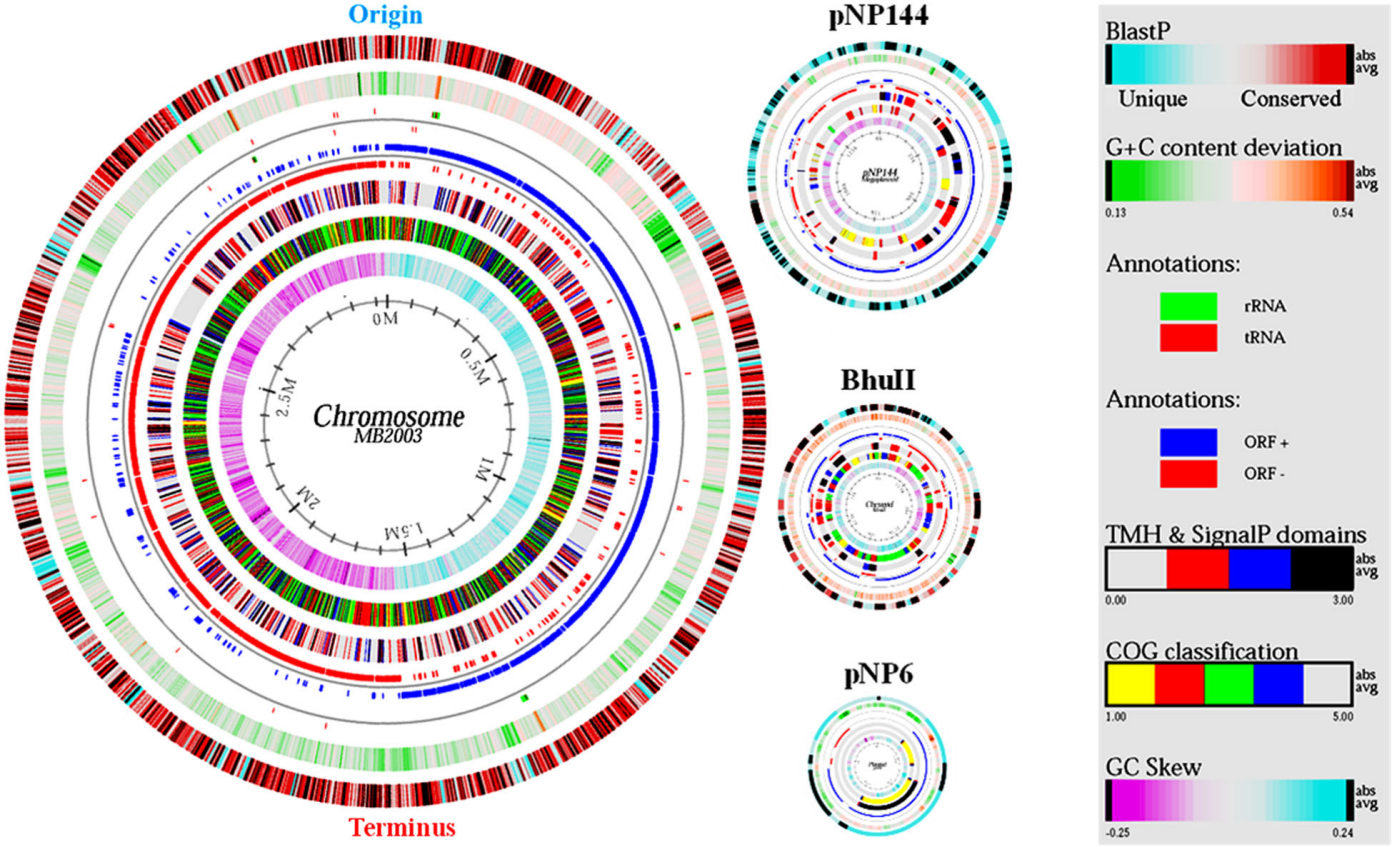
Genome atlas for *B. hungatei* MB2003. The figure represents a circular view of the four replicons that make up the *B. hungatei* MB2003 genome. The key at the right describes the concentric circles within each replicon in the outermost to innermost direction. The diagram was created using GENEWIZ [66] and custom-developed software. The innermost circle 1 shows GC-skew; Circle 2 shows COG classification: predicted ORFs were analysed using the COG database and grouped into the five major categories: yellow, information storage and processing; red, cellular processes and signalling; green, metabolism; blue, poorly characterised; and uncoloured, ORFs with uncharacterized COGs or no COG assignment. Circle 3 shows transmembrane helices (TMH) and SignalP domains: the four categories represent, uncoloured, absent; red, TMH; blue, SignalP; and black, both TMH and SignalP present. Circle 4 shows ORF orientation: ORFs in sense orientation (ORF+) are shown in blue; ORFs oriented in antisense direction (ORF-) are shown in red. Circle 5 shows ribosomal machinery: tRNAs and rRNAs are shown as green or red lines, respectively. Clusters are represented as coloured boxes to maintain readability. Circle 6 shows G + C content deviation from the average: GC-content is shown in either green (low GC spike) or orange (high GC spike). A box filter was applied to visualize contiguous regions of low or high GC deviations. Circle 7 shows BLAST similarities: deduced amino acid sequences were compared against the nonredundant (nr) database using gapped BLASTP [67]. Regions in blue represent unique proteins in MB2003, whereas highly conserved features relative to sequences in the nr database are shown in red. The degree of colour saturation corresponds to the level of similarity. The predicted origin and terminus of DNA replication are indicated

The genome of 10.1601/nm.4132 MB2003 consists of four replicons [21, 31]; a single chromosome (3,143,784 bp, %G + C 39.91), a chromid or secondary chromosome (BhuII, 91,776 bp, %G + C 37.71), a megaplasmid (pNP144, 144,470 bp, %G + C 36.86) and a plasmid (pNP6, 6284 bp, %G + C 35.71). The total size of the closed genome is 3,386,314 bp with an overall %G + C content of 39.71%. A total of 3064 genes were predicted, of which 2983 (97.4%) were protein-coding genes. A putative function was assigned to 2225 of the protein-coding genes, while 775 protein coding genes were annotated as hypothetical proteins. The MB2003 chromosome encodes 2758 genes, and BhuII, pNP144 and pNP6 encode 89, 147 and 6 genes, respectively. The properties and statistics of the MB2003 genome are summarized in Tables 4, 5 and 6. The nucleotide sequences of the MB2003 chromosome, chromid (BhuII), megaplasmid (pNP144) and plasmid (pNP6) have been deposited in Genbank under accession numbers CP017831, CP017830, CP017832 and CP017833. The genome atlas for 10.1601/nm.4132 MB2003 is shown in Fig. 5.

**Table 4:** Summary of MB2003 genome replicon features

Replicon type	Size (bp)	Topology	INSDC identifier	RefSeq ID
Chromosome	3,143,784	circular	CP017831	NZ_CP017831
Chromid_BhuII	91,776	circular	CP017830	NZ_CP017830
Megaplasmid_pNP144	144,470	circular	CP017832	NZ_CP017832
Plasmid_pNP6	6284	circular	CP017833	NZ_CP017833

**Table 5:** MB2003 genome statistics

Attribute	Value	% of total^a^
Genome size (bp)	3,386,314	100
DNA coding (bp)	3,064,986	90.51
DNA G + C (bp)	1,344,683	39.71
DNA scaffolds	4	100
Total genes	3064	100
Protein coding genes	2983	97.36
RNA genes	60	1.96
Pseudogenes	17	0.56
Genes in internal clusters	160	5.22
Genes with function predicted	2247	73.34
Genes assigned to COGs	1842	61.34
Genes with Pfam domains	2350	78.26
Genes with signal peptides	148	4.93
Genes with transmembrane helices	881	29.34
CRISPR repeats	2	

^a^The total is based on either the size of the genome in base pairs or the total number of genes or protein-coding genes in the annotated genome

**Table 6:** Number of genes associated with the general COG functional categories

Code	Value	% of total^a^	Description
J	194	9.52	Translation, ribosomal structure and biogenesis
A	0	0	RNA processing and modification
K	149	7.31	Transcription
L	88	4.32	Replication, recombination and repair
B	0	0	Chromatin structure and dynamics
D	32	1.57	Cell cycle control, Cell division, chromosome partitioning
V	65	3.19	Defense mechanisms
T	139	6.82	Signal transduction mechanisms
M	155	7.61	Cell wall/membrane biogenesis
N	61	2.99	Cell motility
U	22	1.08	Intracellular trafficking and secretion
O	78	3.83	Posttranslational modification, protein turnover, chaperones
C	69	3.39	Energy production and conversion
G	243	11.93	Carbohydrate transport and metabolism
E	177	8.69	Amino acid transport and metabolism
F	80	3.93	Nucleotide transport and metabolism
H	79	3.88	Coenzyme transport and metabolism
I	72	3.53	Lipid transport and metabolism
P	79	3.88	Inorganic ion transport and metabolism
Q	16	0.79	Secondary metabolites biosynthesis, transport and catabolism
R	158	7.76	General function prediction only
S	73	3.58	Function unknown
–	1245	40.33	Not in COGs

^a^The total is based on the total number of protein coding genes in the genome

## Insights from the genome sequence

### Comparison of the MB2003, 10.1601/nm.4132 JK615^T^, and 10.1601/nm.13010 B316^T^ genomes

A comparison of the 10.1601/nm.4132 MB2003 genome with the draft genome of 10.1601/nm.4132 JK615^T^ [32] and the complete 10.1601/nm.13010 B316^T^ genome is shown in Table 7. The MB2003 genome is 8633 bp smaller than JK615^T^ and contains 27 fewer protein-coding genes. Although several plasmid replication genes have been identified in the JK615^T^ draft genome, the presence of extrachromosomal elements requires experimental validation.

**Table 7:** Genome statistics of MB2003, JK615^T^ and B316^T^

Attribute	*B. hungatei* MB2003		*B. hungatei* JK615^Tb^		*B. proteoclasticus* B316^T^	
	Value	% of total^a^	Value	% of total^a^	Value	% of total^a^
Status	Complete		Draft		Complete	
Isolation source	Bovine rumen		Ovine rumen		Bovine rumen	
Genome size (bp)	3,386,314	100	3,394,947	100	4,404,886	100
DNA coding (bp)	3,064,986	90.51	3,108,180	91.55	3,954,077	89.77
DNA G + C (bp)	1,344,683	39.71	1,353,252	39.86	1,762,323	40.01
Number of replicons	4		NA		4	
DNA scaffolds	4	100	22	100	4	100
Total genes	3064	100	3104	100	3863	100
Protein coding genes	2983	97.36	2996	96.52	3739	96.79
RNA genes	60	1.96	55	1.78	68	1.75
rRNA operons	4		4		6	
tRNA genes	48	1.57	46	1.49	50	1.29
Pseudo genes	17	0.56	49		54	1.39
Genes in internal clusters	160	5.22	211	6.82	327	8.43
Genes with function prediction	2225	72.62	2314	74.55	2505	64.85
Genes assigned to COGs	1842	61.34	1861	60.17	2075	53.49
Genes with Pfam domains	2350	78.26	2407	77.82	2784	71.77
Genes with signal peptides	148	4.93	137	4.43	269	6.93
Genes with transmembrane helices	881	29.34	847	27.38	1061	27.35
CRISPR repeats	2		NA		NA	
Reference	This report		[32]		[29]	

^a^The total is based on either the size of the genome in base pairs or the total number of genes or protein-coding genes in the annotated genome. ^b^Indicates draft genome sequence

**Fig. 6:**
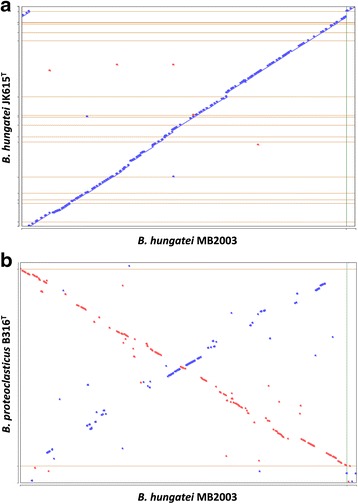
Genome synteny analysis. Alignment of the *B. hungatei* MB2003 genome against the draft genome of *B. hungatei* JK615^T^ (**a**) and the complete genome of *B. proteoclasticus* B316^T^ (**b**). Whenever the two sequences agree, a colored line or dot is plotted. If the two sequences were perfectly identical, a single line would go from the bottom left to the top right. Units displayed in base-pairs. Color codes: blue, forward sequence, red, reverse sequence

A novel feature of both the MB2003 and B316^T^ genomes is the presence of chromids or secondary chromosomes [33]. Chromids are replicons that have %G + C content similar to that of their main chromosome, but have plasmid-type maintenance and replication systems, are smaller than the chromosome, but are usually larger than any other plasmids present. Chromids contain genes essential for growth and maintenance of the organism along with several core genus-specific genes that can be found on the chromosome in other species of bacteria [33]. The Bhu II replicon has most of these characteristics and therefore has been designated as a chromid of MB2003. In B316^T^, almost 10% of the genes encoding enzymes that have a role in carbohydrate metabolism and transport are found on the chromid [29]. The Bhu II chromid of MB2003 also encodes genes with similar predicted functions (Table 9). Since the Bhu II chromid of MB2003 is smaller than the BPc2 chromid of B316^T^ (186,325 bp), it is now the smallest chromid reported for bacteria. Comparison of MB2003, JK615^T^ and B316^T^ genomes based on COG category (Table 8) and synteny analysis (Fig. 6), show that these 10.1601/nm.4129 species and strains are genetically similar. Although the MB2003 and B316^T^ genome sizes differ, the basic metabolism of these two rumen bacterial species is indicated to be similar.

**Table 8:** Comparison of MB2003, JK615^T^ and B316^T^ protein coding gene percentages to COG functional categories

Code	% of total^a^			Description
	MB2003	JK615^T^	B316^T^	
J	9.52	9.33	8.96	Translation
A				RNA processing and modification
K	7.31	7.59	7.30	Transcription
L	4.32	4.59	4.63	Replication, recombination and repair
B				Chromatin structure and dynamics
D	1.57	1.60	1.44	Cell cycle control, mitosis and meiosis
V	3.19	2.90	3.19	Defense mechanisms
T	6.82	6.72	7.47	Signal transduction mechanisms
M	7.61	7.45	8.52	Cell wall/membrane biogenesis
N	2.99	3.29	2.75	Cell motility
U	1.08	1.26	1.14	Intracellular trafficking and secretion
O	3.83	3.63	3.89	Posttranslational modification, protein turnover, chaperones
C	3.39	3.63	3.72	Energy production and conversion
G	11.93	11.99	12.15	Carbohydrate transport and metabolism
E	8.69	8.85	7.91	Amino acid transport and metabolism
F	3.93	3.82	3.98	Nucleotide transport and metabolism
H	3.88	3.77	3.23	Coenzyme transport and metabolism
I	3.53	3.19	2.80	Lipid transport and metabolism
P	3.88	4.06	2.75	Inorganic ion transport and metabolism
Q	0.79	0.68	0.79	Secondary metabolites biosynthesis, transport and catabolism
R	7.76	6.91	7.43	General function prediction only
S	3.58	3.53	4.11	Function unknown
–	40.33	39.83	46.51	Not in COGs

^a^The percentage is based on the total number of protein coding genes in the genome

### Butyrate production

For the production of butyrate and H_2_ from glucose, the MB2003 genome possesses a pyruvate:ferredoxin oxidoreductase gene, *nifJ* (bhn_I2528) required for pyruvate conversion to acetyl-CoA, as well as a butyryl-CoA dehydrogenase/electron transferring flavoprotein *bcd*-*etfAB* (bhn_I2225, bhn_I2221 and bhn_I2222) to generate ATP by classic substrate level phosphorylation. In addition, MB2003 possesses genes that encode all six subunits of the Rnf (*rnfA*, *rnfB*, *rnfC*, *rnfD*, *rnfE*, *rnfG*) and Ech (*echA*, *echB*, *echC*, *echD*, *echE*, *echF*) hydrogenases. These pathways involve the transmembrane ion pumps Ech [34] or Rnf [35–38], that generate a transmembrane proton and/or sodium electrochemical potential from redox cofactors for ATP synthesis by ETP [34, 36]. The MB2003 genome does not possess genes for PorABDG, a pyruvate ferredoxin oxidoreductase similar in function to NifJ or genes for EhaA-R, EhbA-P, HydA-C, MbhLKJ, or MvhADG/HdrABC similar in function to the Fd-dependent Ech hydrogenase. In addition, an alternative pathway exists where formate is predicted to be the end product and involves the decarboxylation of acetyl-CoA by a pyruvate formate lyase *pflB* (bhn_I0124) instead of NifJ. It has been proposed that Ech and Rnf work in concert with NifJ and Bcd-Etf complex to drive ATP synthesis by ETP during glucose fermentation to butyrate [34, 36, 39]. Interestingly, the vast majority of anaerobic prokaryotes appear to possess either an Ech or Rnf but not both [40, 41]. However, a recent analysis of rumen prokaryotic genomes identified 10.1601/nm.4129 and 10.1601/nm.4145 as a rare group of bacteria that possess genes for both Ech and Rnf [42]. These findings warrant further biochemical investigation to determine the activity of Ech and Rnf in 10.1601/nm.4129.

The MB2003 pathways for butyrate production presume the possession of a complete Embden-Meyerhof-Parnas glycolytic pathway. Enolase (*eno*, EC4.2.1.11), converts 2-phospho-D-glycerate to phosphoenolpyruvate in the second to last step of the EMP pathway. Previous work has shown that B316^T^ lacks a detectable enolase [29], and the Methylglyoxal Shunt was proposed as a possible alternative to the EMP pathway. In this pathway the dihydroxyacetone phosphate is transformed to pyruvate via methylglyoxal and d-lactate dehydrogenase, encoded by *ldhA* [43]. The MB2003 genome possesses two methylglyoxal synthase genes, *mgsA* (bhn_I1328 and bhn_I1996), glyoxalase gene *gloA* (bhn_I1783) and an alternative l-lactate dehydrogenase, encoded by *ldh* (bhn_I0363). MB2003 has the same set of genes as B316^T^ for the production of butyrate, formate, acetate and lactate, but also is the only 10.1601/nm.4132 reported to date that lacks a detectable enolase gene. Genome sequences from a wider range of 10.1601/nm.4132 and 10.1601/nm.13010 strains are required to determine if these are common features in these organisms.

### Polysaccharide degradation

The Carbohydrate-Active enZYmes database was used to identify glycoside hydrolases, glycosyl transferases, polysaccharide lyases, carbohydrate esterases and carbohydrate-binding protein module families within the MB2003 genome. MB2003 has a similar CAZyme profile to B316^T^ [21, 31], and analysis of the functional domains of enzymes involved in the breakdown or synthesis of complex carbohydrates, has revealed the polysaccharide-degrading potential of this rumen bacterium.

Approximately 3% of the MB2003 genome (90 CDSs) is predicted to encode either secreted or intracellular proteins dedicated to polysaccharide degradation, similar to that found in B316^T^. The MB2003 genome is predicted to encode 19 secreted (16 GHs, two CEs and one CBP) and 65 intracellular (59 GHs, 5 CEs and one PL) proteins involved in polysaccharide breakdown (Table 9). The enzymatic profiles of MB2003 and JK615^T^ are almost identical, as both possess the same genes encoding predicted secreted and intracellular CAZymes in their genomes (Table 9). Out of the 19 genes predicted to encode secreted polysaccharide degrading enzymes, only two, lysozyme *lyc25B* (bhn_III074) and feruloyl esterase *est1A* (bhn_III076), are encoded by the MB2003 chromid (Bhu II). MB2003 has no secreted enzyme larger than 1000 aa in size, with the average size secreted enzymes being 510 aa. The majority (59) of MB2003 genes involved in polysaccharide breakdown (excluding GTs), had corresponding homologues in B316^T^ and JK615^T^. Three of the genes encoding intracellular proteins were found in the Bhu II chromid: a β-glucosidase *bgl3A* (bhn_III062), a β-galactosidase *bga42A* (bhn_III010) and a polysaccharide deacetylase *est4A* (bhn_III070). The analysis of the Pfam domains from the most abundant GH families (GH2, GH31, GH3, GH13 and GH43) showed they did not contain signal sequences and hence were predicted to be located intracellularly. Similarly, CAZymes with predicted roles in xylan and pectin degradation, the GH8, GH28, GH51, GH67, GH88, GH105, GH115, CE2 and CE10 families were also predicted to be intracellular. Of these, MB2003 contains CAZymes with homologues in B316^T^ except for the α-L-arabinofuranosidase *arf51C* (bhn_I1509). These findings suggest that a variety of complex oligosaccharides resulting from extracellular hydrolysis are metabolized within the cell.

**Table 9:** Genes encoding predicted polysaccharide degrading enzymes in the MB2003 genome

Locus tag	Name	Annotation	Size (aa)	CAZy^a^	Binding domains
bhn_I2518	*bga2A*	β-galactosidase^b^	1034	GH2	
bhn_I0827	*bga2C*	β-galactosidase^b^	714	GH2	
bhn_I1587	*bga2B*	β-galactosidase^b^	825	GH2	
bhn_I0200	*gh2B*	glycoside hydrolase family 2^b^	641	GH2	
bhn_I1127	*gh2A*	glycoside hydrolase family 2^b^	912	GH2	
bhn_I1849	*gh2C*	glycoside hydrolase family 2^b^	776	GH2	
bhn_III062	*bgl3A*	β-glucosidase^b^	803	GH3	
bhn_I0707	*bgl3B*	β-glucosidase^b^	808	GH3	
bhn_I0180	*bgl3C*	β-glucosidase^b^	671	GH3	
**bhn_I0706**	***bgl3D***	**β-glucosidase** ^**b**^	**982**	**GH3**	**C-terminal TMH**
bhn_I0189	*xyl3A*	β-xylosidase^b^	707	GH3	
**bhn_I1640**	***bhx3A***	**β-N-acetylhexosaminidase** ^**b**^	**427**	**GH3**	
**bhn_I1693**	***cel5C***	**endo-1,4-β-glucanase** ^**b**^	**543**	**GH5**	**CBM2a**
**bhn_I0165**	***cel5A***	**endo-1,4-β-glucanase/xylanase** ^**b**^	**417**	**GH5**	
bhn_I1756	*xyn8A*	reducing end xylose-releasing exo-oligoxylanase^b^	383	GH8	
bhn_I0834	*cel9B*	cellodextrinase^b^	552	GH9	CelD
**bhn_I0568**	***xyn10B***	**endo-1,4-β-xylanase** ^**b**^	**425**	**GH10**	
**bhn_I0169**	***xyn10A***	**endo-1,4-β-xylanase** ^**b**^	**451**	**GH10**	
bhn_I1458	*glgB2*	1,4-α-glucan branching enzyme^b^	824	GH13	CBM48
bhn_I0053	*glgB1*	1,4-α-glucan branching enzyme^b^	663	GH13	CBM48
bhn_I2702	*amy13A*	α-amylase^b^	697	GH13	CBM34
**bhn_I0634**	***amy13B***	**α-amylase** ^**b**^	**536**	**GH13**	
bhn_I1680	*amy13C*	α-amylase^b^	434	GH13	
bhn_I0669	*amy13D*	α-amylase^b^	511	GH13	
bhn_I1153	*glgX1*	glycogen debranching enzyme^b^	726	GH13	CBM48
bhn_I1315	*glgX2*	glycogen debranching enzyme^b^	648	GH13	
bhn_I0652	*suc13P*	sucrose phosphorylase^b^	553	GH13	
**bhn_I2526**	***chi18A***	**chitinase** ^**b**^	**567**	**GH18**	
**bhn_I1254**	***lyc25A***	**lysozyme** ^**b**^	**362**	**GH25**	
**bhn_III074**	***lyc25B***	**lysozyme** ^**b**^	**515**	**GH25**	
**bhn_I0191**	***lyc25C***	**lysozyme** ^**b**^	**561**	**GH25**	
**bhn_I1763**	***lyc25D***	**lysozyme** ^**b**^	**242**	**GH25**	
bhn_I0527	*lyc25E*	lysozyme^b^	1213	GH25	Big2 (×2)
bhn_I1287	*aga27A*	α-galactosidase^b^	577	GH27	
bhn_I0082	*gh27A*	glycoside hydrolase family 27^b^	442	GH27	
bhn_I1952	*pg128A*	polygalacturonase^b^	531	GH28	
bhn_I2679	*pgl28B*	polygalacturonase^b^	519	GH28	
bhn_I1087	*fuc29A*	α-L-fucosidase^b^	475	GH29	
**bhn_I2734**	***gh30A***	**glycoside hydrolase family 30** ^**b**^	**575**	**GH30**	
bhn_I1581	*gh31A*	glycoside hydrolase family 31^b^	756	GH31	
bhn_I2191	*gh31C*	glycoside hydrolase family 31^b^	674	GH31	
bhn_I0283	*gh31B*	glycoside hydrolase family 31^b^	635	GH31	
bhn_I0582	*scr32A*	sucrose-6-phosphate hydrolase^b^	493	GH32	
bhn_I0826	*bga35A*	β-galactosidase^b^	622	GH35	
bhn_I1817	*bga35B*	β-galactosidase^b^	735	GH35	
bhn_I0644	*aga36A*	α-galactosidase^b^	782	GH36	
bhn_I1583	*aga36B*	α-galactosidase^b^	620	GH36	
bhn_I1945	*aga36C*	α-galactosidase^b^	730	GH36	
bhn_I0086	*man38A*	α-mannosidase^b^	1053	GH38	
bhn_III010	*bga42A*	β-galactosidase^b^	673	GH42	
**bhn_I0167**	***xsa43A***	**xylosidase/arabinofuranosidase** ^**b**^	**543**	**GH43**	**CBM6**
bhn_I0981	*xsa43B*	xylosidase/arabinofuranosidase^b^	301	GH43	
bhn_I2037	*xsa43C*	xylosidase/arabinofuranosidase^b^	302	GH43	
bhn_I2111	*xsa43D*	xylosidase/arabinofuranosidase^b^	517	GH43	
bhn_I2735	*xsa43E*	xylosidase/arabinofuranosidase^b^	352	GH43	
bhn_I0032	*xsa43G*	xylosidase/arabinofuranosidase^b^	312	GH43	
bhn_I0164	*xsa43F*	xylosidase/arabinofuranosidase and esterase^b^	925	GH43	
bhn_I1509	*arf51C*	α-L-arabinofuranosidase^b^	630	GH51	
bhn_I2228	*arf51A*	α-L-arabinofuranosidase^b^	502	GH51	
bhn_I0010	*arf51B*	α-L-arabinofuranosidase^b^	504	GH51	
**bhn_I0670**	***agn53A***	**arabinogalactan endo-1,4-β-galactosidase** ^**b**^	**439**	**GH53**	
bhn_I0183	*agu67A*	α-D-glucuronidase^b^	662	GH67	
**bhn_I2177**	***mal77A***	**4-α-glucanotransferase** ^**b**^	**506**	**GH77**	
bhn_I0697	*ugl88A*	unsaturated glucuronyl hydrolase^b^	385	GH88	
bhn_I2381	*ugl88B*	unsaturated glucuronyl hydrolase^b^	383	GH88	
bhn_I2196	*cbp94A*	cellobiose phosphorylase^b^	814	GH94	
bhn_I1582	*gh95A*	glycoside hydrolase family 95^b^	734	GH95	
bhn_I2548	*gh105A*	unsaturated rhamnogalacturonyl hydrolase^b^	349	GH105	
bhn_I0090	*gh105B*	unsaturated rhamnogalacturonyl hydrolase^b^	363	GH105	
bhn_I2549	*gnpA*	D-galactosyl-β-1-4-L-rhamnose phosphorylase^b^	722	GH112	
bhn_I0185	*gh115A*	α-glucuronidase^b^	947	GH115	
bhn_I1083	*xyl120A*	xylosidase^b^	861	GH120	
bhn_I1738	*xyl120B*	xylosidase^b^	664	GH120	
**bhn_III076**	***est1A***	**feruloyl esterase** ^**b**^	**351**	**CE1**	
bhn_I1244	*est2A*	acetyl-xylan esterase^b^	372	CE2	
bhn_III070	*est4A*	polysaccharide deacetylase^b^	207	CE4	
**bhn_I0843**	***est4C***	**polysaccharide deacetylase** ^**b**^	**280**	**CE4**	
bhn_I0666	*nagA*	N-acetylglucosamine-6-phosphate deacetylase^b^	371	CE9	
bhn_I1609	*est12A*	carbohydrate esterase family 12^b^	584	CE12	
bhn_I1927	*est12B*	carbohydrate esterase family 12^b^	244	CE12	
bhn_I1926	*pl11A*	polysaccharide lyase^b^	746	PL11	
bhn_I0657	*glgP1*	glycogen phosphorylase^b^	769	GT35	
bhn_I2673	*glgP2*	glycogen phosphorylase^b^	824	GT35	
**bhn_I1848**	**–**	**carbohydrate binding protein** ^**b**^	**983**		**CBM2a (×1), CBM6 (×6)**

^a^CAZy descriptions and classifications compiled from the CAZy database [68]. ^b^Indicates homologues in the *B. hungatei* JK615^T^ draft genome. Genes encoding predicted secreted polysaccharide degrading enzymes are in bold

Growth experiments showed MB2003 to be a metabolically versatile bacterium able to grow on a wide variety of monosaccharides, disaccharides and glycosides (Table 2). However, unlike B316^T^, MB2003 and JK615^T^ were unable to utilize the insoluble substrates pectin and xylan for growth (Table 2). In addition, MB2003, JK615^T^ and B316^T^ are unable to degrade cellulose, however among these organisms, only B316^T^ is able to utilize a range of other insoluble plant polysaccharides. The ability of B316^T^ to breakdown pectin, starch and xylan is predicted to be based on nine large (>1000 aa) cell-associated proteins shown to be significantly up-regulated in B316^T^ cells grown on xylan [44]. These are: α-amylase *amy13A* (bpr_I1087), arabinogalactan endo-1,4-β-galactosidase *agn53A* (bpr_I2041), carbohydrate esterase family 12 *est12B* (bpr_I1204), endo-1,3(4)-β-glucanase *lic16A* (bpr_I2326), pectate lyase *pel1A* (bpr_I2372), pectin methylesterase *pme8B* (bpr_I2473), xylosidase/arabinofuranosidase *xsa43J* (bpr_I2935), endo-1,4-β-xylanase *xyn10B* (bpr_I0026), and the cell wall binding domain-containing protein (bpr_I0264). These proteins contain multiple cell wall binding repeat domains (CW-binding domain, Pfam01473) at their C-termini that are predicted to anchor the protein to the peptidoglycan cell membrane. Among these secreted polysaccharidases, some contain single or combinations of catalytic activities: GH10 (endo-1, 4-β-xylanase, *xyn10B*), GH43 (xylosidase/arabinofuranosidase, *xsa43J*), PL1 (pectate lyase, *pel1A*), CE8 and PL9 (pectin methylesterase, *pme8B*) [45, 46]. Neither MB2003 nor JK615^T^ contain any genes encoding CW-binding domains and are thus are markedly different from B316^T^.

A curious feature of MB2003 was the presence of a single large (983 aa) carbohydrate binding protein (CBP, bhn_I1848), also present in JK615^T^ (EJ23DRAFT_00192). The domain structures of bhn_I1848 and EJ23DRAFT_00192 are unusual, containing six CBM6 (Pfam03422) domains towards the N-terminus and a single C-terminal CBM2a (Pfam00553) domain. In contrast, B316^T^ encodes two CBPs (bpr_I0736 and bpr_I1599) where both contain two CBM2a domains, and bpr_I1599 also contains two CBM6 domains [29]. CBM6 non-catalytic modules characteristically bind xylose and are associated with xylanase activity with ligand specificity for xylan [47, 48]. CBM2 domains, are divided into two sub-families: 2a, that bind to crystalline cellulose even when associated with xylanases [49], and 2b, that bind to xylan [50]. Recent studies have shown that in discrete regions of plant cell walls, initial enzymatic attack of pectin increases the access of CBMs to cellulose [51], effectively loosening the polysaccharide interactions to expose the xylan and xyloglucan substrates [52, 53]. This initial stage in enzymatic saccharification of plant cell walls termed amorphogenesis [54], and is a possible role for such CBPs containing multiple non-catalytic domains. In the rumen, MB2003, B316^T^ and JK615^T^ may secrete these non-catalytic CBPs synergistically with polysaccharide-active enzymes as a mechanism to disrupt the interface between polysaccharides to enhance the rate and extent of plant cell wall degradation.

## Conclusion

The 10.1601/nm.4132 MB2003 genome sequence adds valuable information regarding the polysaccharide-degrading potential present in the genus 10.1601/nm.4129. Genomic comparisons revealed that 10.1601/nm.4132 MB2003 shows a high level of similarity with 10.1601/nm.4132 JK615^T^ and 10.1601/nm.13010 B316^T^ type strains, including genes involved in production of butyrate, formate, acetate and lactate. While MB2003 and JK615^T^ encode a large repertoire of enzymes predicted to metabolize insoluble polysaccharides such as xylan and pectin, they are unable to grow on these substrates and instead appear to be equipped to utilize mainly oligo- and monosaccharides as substrates for growth. Although MB2003 has similar phenotypic characteristics and occupies the same habitat as other 10.1601/nm.4129 species, its genome encodes fewer extracellular polysaccharide degrading enzymes, in particular, those that contain multiple cell wall binding repeat domains. The overall genome similarities, metabolic versatility and differences in the abundance of CAZymes observed in 10.1601/nm.13010 and 10.1601/nm.4132 offers a new view of the genes required for polysaccharide degradation in the rumen. MB2003 appears to occupy a ruminal niche as a secondary degrader of oligosaccharides, in order to coexist with fibre-degrading organisms in this dynamic and competitive environment.

## Additional file

Additional file 1: Table S1. Associated MIGS record for *B. hungatei* MB2003, which links to the SIGS supplementary content website. (DOCX 17 kb)

## Abbreviations

Bp: Base pair(s)
CAZymes: Carbohydrate-Active enZYmes
CBMs: Carbohydrate-Binding Module(s)
CEs: Carbohydrate Esterase(s)
Ech: *Escherichia coli* hydrogenase-3-type hydrogenase
Eha: Energy-converting hydrogenase A
Ehb: Energy-converting hydrogenase B
EMP: Embden-Meyerhof-Parnas
ETP: Electron transport phosphorylation
GHs: Glycoside Hydrolase(s)
GTs: Glycosyl Transferase(s)
Hyd: Ferredoxin hydrogenase
Mbh: Membrane-bound hydrogenase
Mvh/Hdr: Methyl viologen hydrogenase/heterodisulfide reductase
Pfl: Pyruvate formate lyase
PLs: Polysaccharide Lyase(s)
Por: Pyruvate ferredoxin oxidoreductase
Rnf: *Rhodobacter* nitrogen fixation
